# Risk factors of frailty/sarcopenia in community older adults: Meta-analysis

**DOI:** 10.1515/med-2025-1259

**Published:** 2025-09-12

**Authors:** Xiaoyang Yan, Jiansong Zhou, Xia Cao

**Affiliations:** Health Management Center, The Third Xiangya Hospital of Central South University, 410013, Changsha, China; Department of Psychiatry, National Clinical Research Center for Mental Disorders, and National Center for Mental Disorders, The Second Xiangya Hospital of Central South University, 410011, Changsha, China; Health Management Center, The Third Xiangya Hospital of Central South University, No. 138, Tongzipo Road, 410013, Changsha, China

**Keywords:** frailty, sarcopenia, prevalence, risk factors, elderly in the community

## Abstract

**Objective:**

This study aimed to review the rates of physical frailty and sarcopenia, as well as associated risk factors, among adults aged 60 and older living in the community.

**Methods:**

A systematic literature search was conducted in PubMed, Wiley Library, Embase, Web of Science, and Cochrane Library for articles published from January 2000 to December 2024. The review followed PRISMA guidelines for meta-analysis.

**Results:**

Sixteen studies with 41,765 participants were included in the meta-analysis. The pooled prevalence of frailty/sarcopenia was 27% (95% CI: 19–35%). Specifically, the pooled prevalence of frailty was 25% (95% CI: 16–38%) and that of sarcopenia was 23% (95% CI: 13–37%). Age, malnutrition, depression, falls, and hypertension were identified as significant risk factors for frailty/sarcopenia.

**Conclusion:**

Nearly 30% of older adults in the community are affected by frailty/sarcopenia. Early identification of these risk factors is crucial for the prevention and management of frailty/sarcopenia. Further research is needed to determine effective interventions and strategies to reduce the incidence of frailty/sarcopenia.

## 1 Introduction

Nowadays, the world is facing a significant challenge of human aging. Various conditions, including frailty [1], sarcopenia [2], cognitive impairment [3], dementia [4], and cancer [5], are associated with human degeneration. Frailty and sarcopenia are two geriatric syndromes with partly overlapping phenotypes, and sarcopenia typically manifests prior to the onset of frailty [6]. Unlike other conditions related to specific abnormality, frailty/sarcopenia is the outcome of multiple etiological factors linked to decreased life satisfaction, disability, and mortality in the older adults [7], leading to a substantial economic burden for individuals and healthcare services [8]. Although frailty/sarcopenia has a high prevalence among older adults worldwide, there is considerable variation in prevalence among different regions and populations [9,10]. A recent meta-analysis revealed that the prevalence of frailty in hospital settings was 47.3%, while in community settings, the pooled prevalence was 18.8% [11]. Another study comparing the prevalence of sarcopenia using different definitions indicated that the prevalence of sarcopenia ranged from 0.7 to 16.8% in a large multinational European population of community-dwelling older adults [12].

Given that frailty/sarcopenia are syndromes influenced by complex factors, it is widely acknowledged that age, female sex, malnutrition, smoking, depression, education years, overweight, chronic disease, hypertension, and alcohol are significant contributory factors to the development of frailty/sarcopenia [13,14,15]. However, the relationship between frailty/sarcopenia and risk factors varies across studies, exhibiting significant diversity in research objectives, methodologies, and diagnostic criteria. Further research is required to fully grasp the impact of these factors. As global interest in healthy aging continues to increase, a more comprehensive understanding of the prevalence of frailty/sarcopenia and risk factors could contribute to advancing discussions on the preservation of functional capacity in older populations. Considering this gap, this study aimed to systematically assess the prevalence and risk factors of frailty/sarcopenia among community-dwelling adults.

## 2 Method

### 2.1 Protocol

This review followed PRISMA guidelines for meta-analysis [16]. All articles were found online, so institutional review board approval was not needed. The protocol is registered in PROSPERO (CRD42023488583).

### 2.2 Search strategies

We conducted searches in several databases, including PubMed, Wiley Library, Embase, Web of Science, and the Cochrane Library, for studies in the period from January 2000 to December 2024 with English restrictions. Specific search terms and formulas were used to ensure search accuracy, including keywords of frailty, sarcopenia, geriatric syndrome, prevalence, risk factors, community-dwelling elderly, elderly health, epidemiology, and the search formula used is: (“frailty” OR “sarcopenia” OR “geriatric syndrome”) AND (“prevalence” OR “epidemiology”) AND (“risk factors”) AND (“community-dwelling elderly” OR “elderly health”).

### 2.3 Inclusion and exclusion criteria

The inclusion criteria were as follows: (1) community-dwelling older adults aged 60 years or above; (2) cross-sectional studies and cohort studies, with English restrictions; and (3) studies reported the prevalence and risk factors associated with frailty/sarcopenia, providing raw data and sufficient information for analysis. The exclusion criteria were as follows: (1) reviews, case reports, opinion articles, and editorial reviews; (2) studies with poor methodological quality and incomplete data reporting; (3) studies that specialize in a specific disease or condition; and (5) studies that did not report relevant prevalence or risk factor data.

### 2.4 Study selection and data extraction

Two investigators (X.Y. and S.H.L.) independently screened records based on the title, abstract, and full texts, after deleting duplicated articles using Endnote 21. Where disagreements arose, they would be settled through consensus with a third reviewer. Data extraction was carried out by three authors (X.Y., Y.Y.Y., and X.C.), who checked each other's results and discussed any disagreements. The following information was collected from individual articles: study details (author, year of publication, country, and study design), characteristics (sample size and participant demographics), the definition/diagnosis criteria of frailty/sarcopenia, prevalence rates, risk factors, and other related results.

### 2.5 Study quality assessment

The quality of the included cross-sectional studies was assessed by two investigators (J.S.Z. and X.C.) using the Agency for Healthcare Research and Quality (AHRQ) scale, covering aspects such as study design, sample representativeness, data collection methods, and result reporting. Each literature was assessed by two independent reviewers. Any discrepancies in quality assessment scores were resolved through discussion or consultation with a third reviewer.

### 2.6 Statistical analysis

A comprehensive statistical method was conducted using R4.3.1 software. First, prevalence data were combined using a random-effects model. To assess the degree of heterogeneity between studies, an *I* ^2^ statistic was calculated, with values ranging from 0 to 100%, with higher *I* ^2^ values indicating more significant heterogeneity. We also performed a broad subgroup analysis based on country and region. Publication bias was evaluated by Begg and Egger regression tests [17]. In addition, sensitivity analysis was conducted to evaluate the robustness of the results from the meta-analysis. For risk factors, odds ratios (OR) and 95% confidence intervals (CI) were calculated to determine the strength of associations between each factor and frailty/sarcopenia. Two-tailed *P* < 0.05 was considered statistically significant.

## 3 Results

### 3.1 Selection process and study characteristics

**Figure 1:**
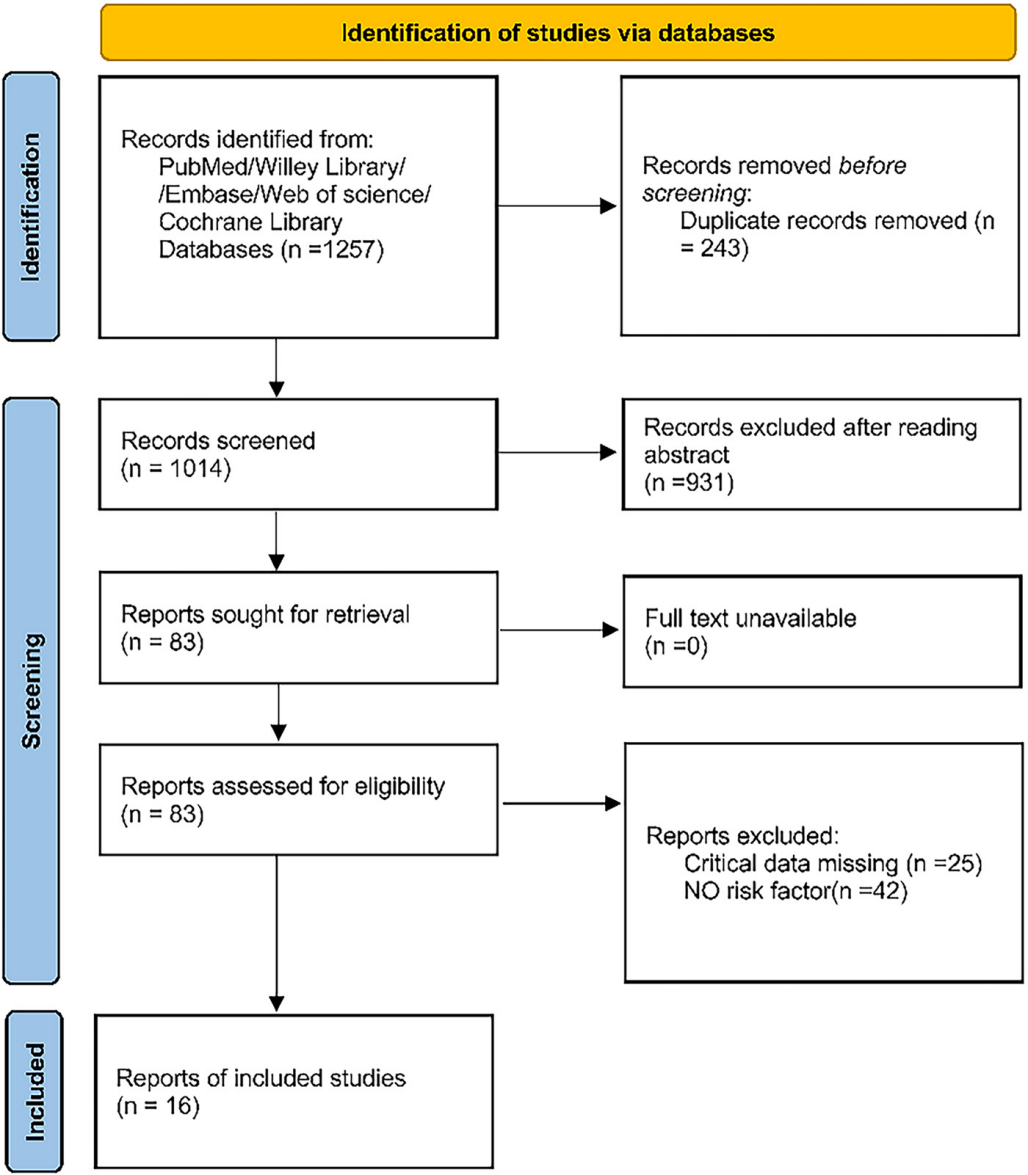
Flow diagram of study screening.

Figure 1 illustrates a selection process with the number of studies at each stage. At first, 1,257 articles were identified. After removing duplicates, 1,014 were screened. Of them, 931 were excluded after the review of titles and abstracts, thus leaving 83 articles for full-text screening. Of these, 25 records were without critical data, and 42 records were without risk factors. This gave a total of 16 studies for review and analysis. The characteristics of the included studies are summarized in Table 1. Most of the studies were from Asia, with five from China [15,18,19,20,21], seven from other Asian countries [22,23,24,25,26,27,28], and four from other countries [29,30,31,32]. Studies were published between 2009 and 2022, with sample sizes ranging from 158 to 28,323 participants. The mean or median age ranged from 62.9 to 78 years, and four studies did not provide the detailed mean or median age for the total sample [19,21,26,28]. Only cross-sectional studies were included.

**Table 1:** Study characteristics

Author	Year of publication	Site	Study design	Sample size	Age (mean, years)	Female proportion (%)	Married proportion (%)	Smoking proportion (%)	Drinking proportion (%)	Years of education	Risk factors
Coelho	2017	Portugal	CS	252	79.2 (7.3)	75.8	19.4	—	—	4.4 (3.6)	1, 2, 3, 5
de Amorim	2019	Brazil	CS	258	62.9 (2.7)	42.2	61.1	50.8	10.5	—	1, 2, 3, 10, 11
Gao	2015	China	CS	329	70.4 (6.8)	58.4	81.2	12.5	10	—	1, 2
Hwang	2022	Korea	CS	1,293	77.98 (1.927)	58.88	—	66.5	23.5	—	4
Kim	2015	Japan	CS	325	78 (2.6)	—	—	—	—	—	1, 8, 9
Liao	2018	China	CS	6,320	>65	61.5	76.3	—	—	—	4
Ma	2009	America	CS	230	77 (7)	82	—	—	—	—	1, 7
Norazman	2020	Malaysia	CS	301	67.1 (5.5)	69.4	52.8	—	—	—	1, 4, 6
Seesen	2021	Thailand	CS	373	70.45 (5.4)	58.4	63.8	—	10.2	—	1, 2, 5, 6, 10, 11
Setiati	2021	Indonesia	CS	908	>60	51.8	71.5	—	—	—	6, 7, 8
Shafiee	2020	Iran	CS	2,462	69.34 (6.4)	51.9	76.8	—	—	—	1, 3, 4
Simsek	2019	Turkey	CS	28,323	>65	—	—	—	—	—	1, 3, 5, 6, 11
Sousa	2012	Brazil	CS	391	74 (6.5)	61.4	35	—	—	—	2, 5, 7, 9
Wang	2019	China	CS	947	68.78 (6.52)	50.89	—	28	14.4	—	1, 2, 5, 6, 7, 10, 11
Wu	2021	China	CS	3,796	66.49 (5.35)	48.5	—	36.4	47.7	—	1, 8, 9, 10, 11
Xing	2022	China	CS	158	>60	58.7	—	63.8	23.4	—	11

Risk factors: 1 age; 2 gender (female); 3 total years of education; 4 overweight; 5 underlying diseases; 6 malnutrition; 7 depression; 8 falls; 9 hypertension; 10 alcohol consumption; 11 smoking. CS: cross-sectional.

### 3.2 Methodological quality

To ensure the validity and dependability of the findings from the cross-sectional studies incorporated in this meta-analysis, we employed the AHRQ standard scale to systematically assess the quality of these studies. Upon thorough examination, it was observed that the majority of the cross-sectional studies analyzed demonstrated satisfactory performance on the AHRQ scale with regard to sample representation, data collection standardization, and result dissemination. Further details are summarized in Table 2.

**Table 2:** Included literature quality evaluation (AHRQ scale)

Author	Year	Rigor of study design	Sample representation	Data collection	Data analysis	No risk of bias	Result reliability
Coelho	2017	Yes	Yes	Yes	Yes	Yes	Yes
de Amorim	2019	Yes	Yes	Yes	Unclear	Yes	Yes
Gao	2015	Yes	Yes	Yes	Yes	Yes	Yes
Hwang	2022	Yes	Yes	Yes	Unclear	Unclear	Yes
Kim	2015	Yes	Yes	Yes	Yes	Yes	Yes
Liao	2018	Yes	Unclear	Yes	Yes	Yes	Yes
Ma	2009	Yes	Yes	Yes	Unclear	Yes	Yes
Norazman	2020	Yes	Yes	Yes	Yes	Unclear	Yes
Seesen	2021	Yes	Yes	Yes	Yes	Yes	Yes
Setiati	2021	Yes	Yes	Yes	Unclear	Yes	Yes
Shafiee	2020	Yes	Yes	Yes	Yes	Unclear	Yes
Simsek	2019	Yes	Yes	Yes	Yes	Yes	Yes
Sousa	2012	Yes	Yes	Yes	Yes	Yes	Yes
Wang	2019	Yes	Yes	Unclear	Unclear	No	Yes
Wu	2021	Yes	Yes	Yes	Yes	Yes	Yes
Xing	2022	Yes	Yes	Yes	Yes	No	Yes

### 3.3 Prevalence of frailty/sarcopenia

**Figure 2:**
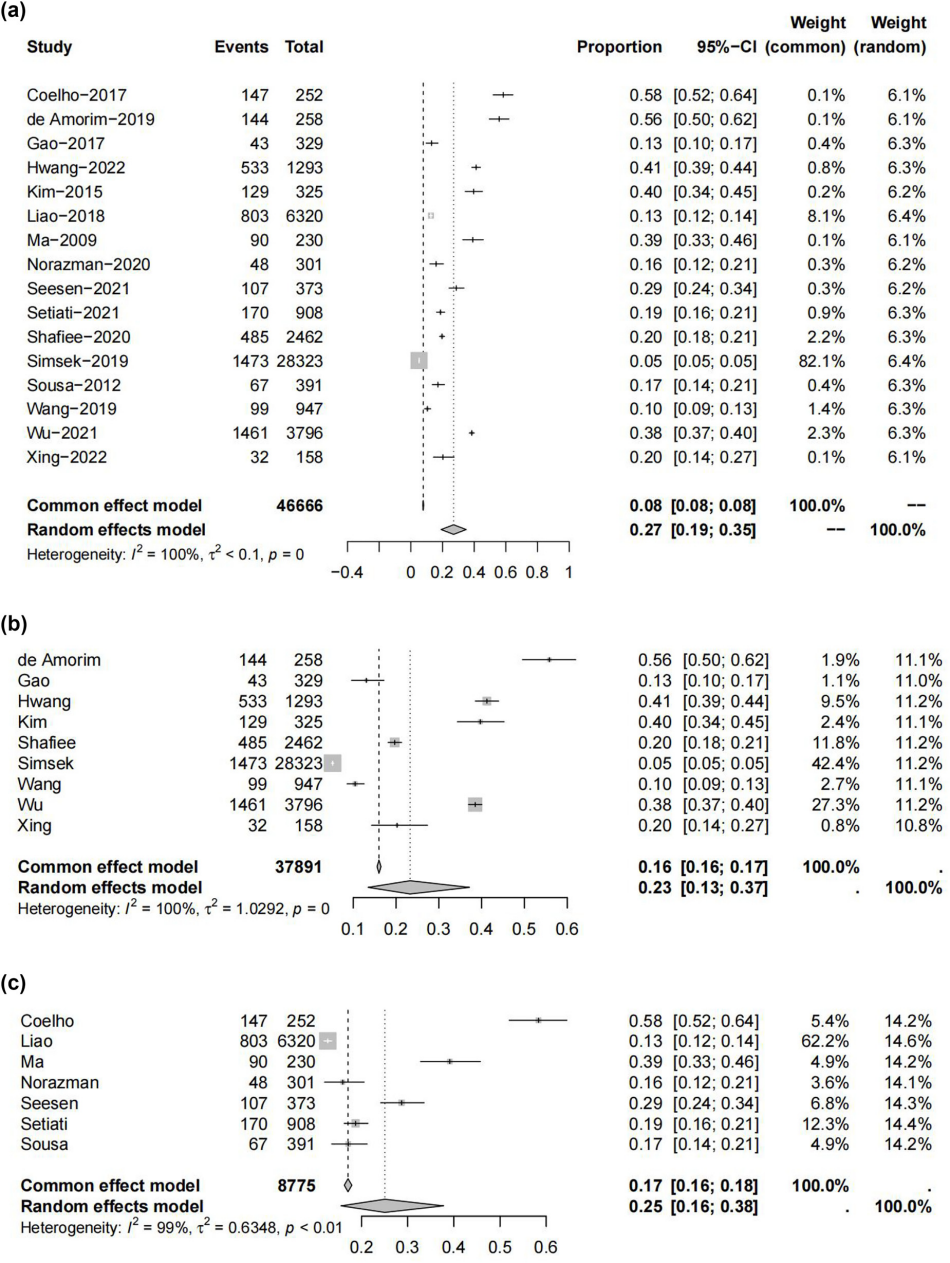
The prevalence of frailty/sarcopenia: (a) total prevalence of frailty/sarcopenia, (b) prevalence of sarcopenia, and (c) prevalence of frailty.

The prevalence of frailty/sarcopenia in individual studies ranged from 5 to 58%. In general, the pooled prevalence of frailty/sarcopenia was 27% (95% CI: 19–35%, *I* ^2^ = 100%, *P* < 0.001). Among these, the pooled prevalence of frailty (7 studies; 8,775 participants) was 25% (95% CI: 16–38%), and the pooled prevalence of sarcopenia (9 studies; 37,891 participants) was 23% (95% CI: 13–37%) (Figure 2). When categorized by country, the incidence of frailty/sarcopenia was 27% in China and 31% in other countries. When categorized by region, the prevalences of frailty/sarcopenia in ascending order in Asia, Europe, and America were 23% (95% CI: 17–30%), 32% (95% CI: 0–84%), and 37% (95% CI: 15–59%), respectively (Table 3).

**Table 3:** Subgroup analysis of the prevalence of frailty/sarcopenia

		Number of literatures	Number of samples	Number of diagnoses	Heterogeneity test		Model	Results of meta-analysis
Characteristics					*I* ^2^(%)	*P*		
Country							Random utility model	
	China	5	11,550	2,438	100	0	Random utility model	0.27 (0.09–0.29)
	Others	11	35,116	3,393	100	<0.01	Random utility model	0.31 (0.21–0.41)
Region							Random utility model	
	America	3	879	294	100		Random utility model	0.37 (0.15–0.59)
	Europe	2	28,575	1,620	100		Random utility model	0.32 (0.00–0.84)
	Asia	11	17,212	3,920	100		Random utility model	0.23 (0.17–0.30)

### 3.4 Factors related to frailty/sarcopenia

The systematic review encompassed 16 articles that collectively identified 11 factors linked to frailty/sarcopenia among older adults residing in community settings, including age, sex, years of education, overweight, underlying disease, malnutrition, depression, falls, hypertension, alcohol consumption, and smoking, which were used for pooled analysis. Among them, the association of frailty/sarcopenia with age, years of education, overweight, underlying disease, alcohol consumption, and smoking did not reach statistical significance. The results of the risk factors analysis are listed in Figure S1.

### 3.5 Publication bias and sensitivity analysis

The results of the publication bias and sensitivity analysis are summarized in Table S1. Only the comparison of overweight and smoking in the meta-analysis, a publication bias, was detected by Begg’s test (*P* = 0.03) and Egger’s test (*P* = 0.04), respectively. This indicated that the possibility of publication bias in the included literature was relatively small. Sensitivity analysis showed the results were stable after excluding any one of the included studies, suggesting that the study results were consistent.

## 4 Discussion

This systematic review and meta-analysis found a pooled prevalence of 27% for frailty/sarcopenia, with frailty at 25% and sarcopenia at 23%, slightly higher than previous reports [9]. Differences in study populations and diagnostic methods may explain the varying prevalence rates. Our meta-analysis included one study focused solely on elderly individuals with hypertension in the community. This could result in a marginally higher estimated pooled prevalence. The incidence of frailty/sarcopenia was 27% in China and 31% in other countries. This can be attributed to factors like race, body size, culture, diet, and the quality of life of the elderly [33]. For instance, Tai chi, as a kind of Chinese traditional exercise, was found to be effective in dealing with frailty/sarcopenia [34].

This study analyzed 11 risk factors, and those found to be significantly associated with frailty/sarcopenia were age, malnutrition, depression, falls, and hypertension. Specifically, the prevalence of frailty/sarcopenia increases with increasing age. Increasing age may lead to changes in muscle mass, composition, and contractile performance, leading to changes in muscle strength and function, and ultimately resulting in increased mobility degradation, increased disability, increased risk of falling, and increased frailty/sarcopenia [35].

Malnutrition is considered to be associated with frailty/sarcopenia. At present, numerous studies have found that malnutrition and sarcopenia have similar pathophysiological mechanisms, and it is one of the important risk factors for sarcopenia [36]. A low energy intake (EI) may lead to not only the loss of fat storage but also muscle mass. A recent study discovered that low suitable EI had a higher negative effect than excessive EI, particularly on physical frailty [37]. Particularly, protein synthesis is significantly related to skeletal muscle mass. A cross-sectional study carried out on 1,490 participants found that protein intake was considered to be a risk factor for frailty [38]. Maintaining a variety of dietary protein sources was recommended in Chinese research of 2,216 subjects [39]. There is an impairment in the anabolic response in the elderly, even with diets rich in protein, and this is called anabolic resistance [40]. To explain the decrease in protein synthesis in the elderly, a previous study has indicated that these changes in gene expression occur at the level of transcription and translation [41]. For instance, the 5′-TOP mRNAs are related to several components of protein synthesis machinery, and their translation efficiency is decreased with aging [41]. These mRNAs are regulated by the mTOR/4E-BP axis, a signaling pathway associated with aging [42]. In addition, another study recently utilizing data from the National Health and Nutrition Examination Survey 2007–2018 included 11,529 participants and found that people with high dietary intake of live microbes had a significantly lower risk of frailty than those with low dietary intake of live microbes [43].

In this study, depression is positively related to frailty/sarcopenia. Previous studies have indicated that depression may be an early indicator of frailty/sarcopenia in older adults, emphasizing the importance of early identification and treatment of depression as part of comprehensive strategies to prevent sarcopenia [44,45]. Besides, bidirectional causal relations between depression and frailty/sarcopenia have been confirmed, including predictive effects both in initial levels and rates of change [46,47]. There are some explanations for the correlation between depression and frailty/sarcopenia. On the one hand, we could say that people with depression are more likely to develop related behavior habits, such as decreased physical activity and medication compliance. These may lead to frailty/sarcopenia, too [48]. On the other hand, frailty/sarcopenia means low levels of physical activity and poor physical functioning, which are risk factors for depression [49]. Levels of inflammatory biomarkers like tumor necrosis factor α and interleukin 6 are relevant to frailty/sarcopenia. These factors are also found to be predictive of depressive symptoms and suicidal ideation and behavior [50]. In addition, changes in the hypothalamic pituitary adrenal axis and chronic inflammation are common in frailty/sarcopenia and depression [51,52]. In genetic connection, a study using 139 pairs of dizygotic twins data in China found that potential genetic correlations, SNPs, genes, and pathways were shared between depression and grip strength, and the latter serves as an indicator of sarcopenia [53]. Also, the frailty GWAS statistics reported 14 genome-wide significant risk loci, and two loci also showed significant association with depression [54].

Falls are significantly related to frailty/sarcopenia. Since falling is the third cause of chronic disability, most previous studies have shown that frailty/sarcopenia represents a significant risk factor for falls in older adults [55–57]. Falls are related to multiple system impairments, especially of muscle mass, balance, and cognition, so could be understood as results of frailty/sarcopenia syndrome [58]. Moreover, falls not only directly lead to physical impairment, but may also be a marker of other potential health problems such as balance disorders, muscle weakness, or neurological dysfunction. Multiple factors contribute collectively to frailty/sarcopenia and fall, including social isolation, elder abuse, inability to prepare and cook meals or to feed oneself and shop, thyroid disease, cardiac failure, and so on [59]. Therefore, the prevention of falls in the elderly is important for the prevention of frailty/sarcopenia.

This study suggests that hypertension is an important risk factor in the elderly population and may increase the risk of frailty/sarcopenia due to its effects on physical multisystem function, including but not limited to cognitive decline, restricted motor capacity, etc. Previous studies, mostly involving community-dwelling old adults, focused on the influence that frailty/sarcopenia has on the management of hypertension [60]. It is generally recognized that assessing the frailty level before developing treatment plans could improve and predict short- and long-term complications of therapeutic strategies [61]. More studies are needed to discuss the causality between frailty/sarcopenia and hypertension. A previous study has found that hypertension was more prevalent in frail elderly patients and was significantly associated with frailty [62], and older adults with both hypertension and frailty have a relatively higher mortality risk [63]. Another multi-center cross-sectional study revealed that hypertension was an independent predictor of sarcopenia in males, and angiotensin-converting enzyme inhibitors showed higher muscle function values [64]. Several pathological mechanisms can account for the association. For example, as a result of aging, chronic inflammation and the production of cytokines are relevant to aging, leading to the onset of frailty/sarcopenia and aging-related diseases like hypertension [65]. Insulin resistance increases the risk of hypertension, and this can also lead to skeletal muscle loss and sarcopenia [66]. Therefore, the early assessment of hypertension is meaningful for elderly people.

This meta-analysis is a reliable overview of the evidence about frailty/sarcopenia. In our study, we retrieved the literature comprehensively and carefully selected them, providing 16 cross-sectional studies about frailty/sarcopenia and its risk factors. Through comprehensive analyses, subgroup analyses, sensitivity analyses, and tests for publication bias, our study has confirmed the robustness and validity of the study findings. In addition, focusing on community-dwelling samples enhances the generalizability of our findings. However, our findings still have some limitations. First, there is significant heterogeneity among parts of the studies that can be explained by the demographic differences across studies, and the reliability of our findings is increased by the low-to-moderate risk of methodological bias. Second, the international sample exhibited an imbalance, with a notable disparity in the number of studies conducted in Asia compared to other countries. Due to the limited studies per country or region, it is difficult to determine how this may impact the results. Third, different tools used to identify frailty/sarcopenia may cause variations in the prevalence. For example, the prevalence of sarcopenia was found to be higher using AWGS compared to the SARC-F and EWGSOP tools [9]. Therefore, the inclusion of studies using various diagnostic approaches may influence the outcomes of our research.

## 5 Conclusion

The community-dwelling older adults are prone to suffering from frailty/sarcopenia, and age, malnutrition, depression, falls, and hypertension are identified as risk factors for frailty/sarcopenia. These findings highlight the importance of early detection of these risk factors for preventing and intervening in frailty/sarcopenia. Additional research is necessary through prospective clinical trials or cohort studies to evaluate the most effective interventions and strategies for reducing the incidence of frailty/sarcopenia.

## Supplementary Material

Supplementary material
