# Supplementary material for: Risk factors of frailty/sarcopenia in community older adults: Meta-analysis
Source: Open Med (Wars). 2025 Sep 12;20(1):20251259. doi: 10.1515/med-2025-1259 (PMC12452068; doi:10.1515/med-2025-1259)
Supplement: Supplementary material [file med-2025-1259-sm.pdf]

Supplementary material

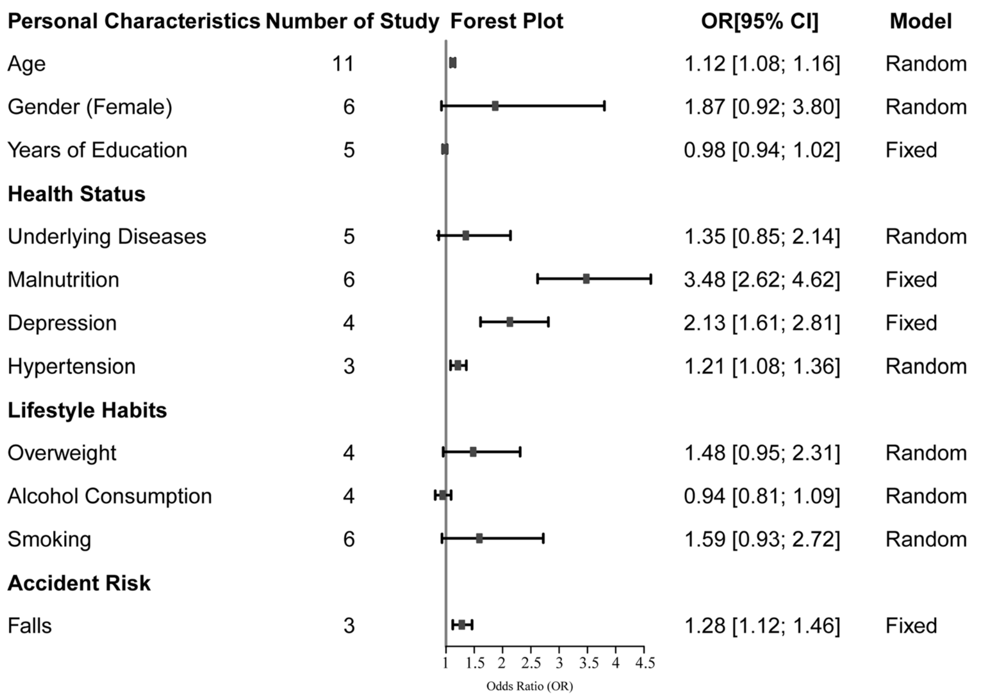

Figure S1: Forest plot of frailty/sarcopenia among community-dwelling older adults and associated risk factors.

Table S1: Publication bias assessment (Begg's test and Egger's test)

	Begg's test		Egger's test	
	***Z* score**	***P* valve**	***t* score**	***P* valve**
Age	0.86	0.39	1.35	0.21
Sex (Female)	1.69	0.09	1.17	0.31
Years of Education	1.96	0.05	2.28	0.11
Overweight	5.33	**0.03**	0.52	0.6
Underlying disease	0.49	0.62	1.04	0.49
Malnutrition	2.04	0.11	0.94	0.35
Depression	0.68	0.49	−0.11	0.92
Falls	0.52	0.6	0.58	0.66
Hypertension	−0.52	0.6	−0.55	0.68
alcohol consumption	−0.68	0.5	−0.51	0.66
smoking	0.56	0.57	3.07	0.04
